# Adapting the design of a Web-based decision support clinical trial during the COVID-19 pandemic

**DOI:** 10.1186/s13063-021-05700-z

**Published:** 2021-10-23

**Authors:** Jessica Meline, Jason M. Prigge, Debbie Dye, Julie Rieder, Onur Asan, Sumedha Chhatre, Liana Fraenkel, Jeffrey D. Kravetz, Keri L. Rodriguez, Jeff Whittle, Dana Kaminstein, Marilyn M. Schapira

**Affiliations:** 1 The Center for Health Equity Research and Promotion (CHERP) at the Michael J. Crescenz Veterans Affairs (VA) Medical Center, 3900 Woodland Ave, Philadelphia, PA 19104 USA; 2 Research Service at Clement J. Zablocki VA Medical Center, 5000 W National Avenue, Milwaukee, WI 53295 USA; 3 Clinical and Translational Science Institute, Medical College of Wisconsin, 8701 W. Watertown Plank Rd, Milwaukee, WI 53226 USA; 4 Center for Advancing Population Science, Medical College of Wisconsin, 8701 W. Watertown Plank Rd, Milwaukee, WI 53226 USA; 5 The Stevens Institute of Technology, School of Systems and Enterprises, 1 castle terrace point, Hoboken, NJ 07030 USA; 6 The Department of Psychiatry, the University of Pennsylvania Perelman School of Medicine, 3535 Market St., Suite 4051, Philadelphia, PA 19104 USA; 7 Yale University School of Medicine, 20 York St., New Haven, CT 06510 USA; 8 VA Connecticut Healthcare System, 950 Campbell Ave, West Haven, CT 06516 USA; 9 Organizational Dynamics, Liberal and Professional Studies, School of Arts & Sciences, University of Pennsylvania, 3400 Market St. Suite 100, Philadelphia, PA 19104 USA; 10 Division of General Internal Medicine, University of Pennsylvania Perelman School of Medicine, 1316 Blockley Hall, 423 Guardian Dr, Philadelphia, PA 19104 USA

**Keywords:** COVID-19, Shared decision-making, Lung cancer screening, Clinical trial

## Abstract

**Background:**

The public health crises that emerged in the COVID-19 pandemic significantly impacted the provision of medical care and placed sudden restrictions on ongoing clinical research. Patient-facing clinical research confronted unique challenges in which recruitment and study protocols were halted and then adapted to meet safety procedures during the pandemic. Our study protocol included the use of a Lung Cancer Screening Decision Tool (LCSDecTool) in the context of a primary care visit and was considerably impacted by the pandemic.

We describe our experience adapting a multi-site clinical trial of the LCSDecTool within the Department of Veterans Affairs Health Care System. We conducted a randomized controlled trial (RCT) comparing the LCSDecTool to a control intervention. Outcomes included lung cancer screening (LCS) knowledge, shared decision-making, and uptake and adherence to LCS protocol. We identified three strategies that led to the successful adaptation of the study design during the pandemic: (1) multi-level coordination and communication across the organization and study sites, (2) flexibility and adaptability in research during a time of uncertainty and changes in regulation, and (3) leveraging technology to deliver the intervention and conduct study visits, which raised issues concerning equity and internal and external validity.

**Conclusion:**

Our experience highlights strategies successfully employed to adapt an intervention and behavioral research study protocol during the COVID-19 pandemic. This experience will inform clinical research moving forward both during and subsequent to the constraints placed on research and clinical care during the COVID-19 pandemic.

## Background

We report the procedures taken to adapt a randomized controlled trial (RCT) of a Web-based decision support tool (LCSDecTool) designed for Veterans eligible for lung cancer screening (LCS). The LCSDecTool was designed to inform patients and support a shared decision-making process [1, 2]. We describe our experience throughout the pandemic, highlighting effective coordination that allowed the study to continue, the importance of flexibility in research methods and workforce during times of uncertainty, and the experience transitioning to virtual methods to deliver intervention and conduct study visits. We also identify barriers that these processes may create in providing equity in participation in clinical research and validity of research findings among diverse populations.

The COVID-19 pandemic that emerged in early 2020 placed sudden and significant restrictions on ongoing clinical research. To ensure the safety of healthcare workers, patients, and family members, healthcare systems in the USA and internationally implemented emergency procedures to eliminate non-essential in-person contacts. Restrictions extended to health research conducted in both laboratory and clinical settings [3–5]. Clinical research involving patient subjects required immediate changes in protocols [6]. In many cases, study recruitment and face-to-face study visits were halted, requiring research teams to manage resources, adapt study designs, and plan for implementing modified protocols when permitted by the health care system and/or academic center overseeing the research [5]. This process was particularly challenging for behavioral clinical trials involving face-to-face interventions in the context of a clinical visit.

The COVID-19 pandemic presents unique challenges to clinical research focused on shared decision-making as this process often occurs in the clinical setting of a primary care visit. During the pandemic, most primary care visits shifted from face-to-face to virtual care to follow social distancing guidelines to keep patients, staff, and family members safe. Recommendations for primary care providers included conducting initial visits through video/telephone and limiting the number of face-to-face visits [7, 8]. Research protocols incorporating the primary care visit, including those with shared decision-making interventions, had to adapt in tandem with the delivery of clinical care during the pandemic.

Our study, *Incorporating Veterans’ Preferences into Lung Cancer Screening Decisions*, started recruitment on 1/8/19. In March of 2020, we had recruited approximately 50% of our sample size goal of 200 patient participants. On 3/17/20, due to the COVID-19 pandemic, study recruitment and in-person research activities were halted by the Office of Research and Development (ORD) at the Department of Veterans Affairs.

The goal of our study was to evaluate the efficacy of the LCSDecTool versus a control intervention on shared decision-making and uptake and adherence to LCS protocols. The LCSDecTool was designed for use across multiple devices (tablet, computer, mobile phone) and for self-navigation. We hypothesized that those who used the LCSDecTool would have greater knowledge, decreased decisional conflict, and decreased uptake of LCS than a control population. The target population was Veterans receiving primary care at a participating Veterans Affairs (VA) Medical Center and who met the criteria for LCS according to the 2013 guidelines of the United States Preventive Services Task Force (USPSTF). These criteria include age 55 to 80, a smoking history of at least 30 pack years, and being either currently smoking or having quit within 15 years. The USPSTF specifically recommends that eligible patients undergo shared decision-making before receiving LCS.

### Original study protocol

The current phase of this study is an RCT of the LCSDecTool compared to a control intervention [9]. Participants were recruited to the RCT by mailed letter and a follow-up telephone call. Research coordinators met with study participants for the in-person baseline visit (T0) prior to primary care appointments. This design allowed participants the opportunity for a shared decision-making discussion with their clinician about LCS after using the LCSDecTool or control intervention. During the T0 visit, participants completed written informed consent and Health Insurance Portability and Accountability Act (HIPAA) authorization and baseline surveys and used an iPad device to view either the experimental (LCSDecTool) or the control (general information on cancer screening) intervention. Randomization occurred automatically when the participant logged onto the website with their de-identified number. While participants viewed experimental or control content, coordinators recorded field note observations to capture impressions of the interventions and usability data, such as navigational issues. Following T0, participants attended their in-person primary care appointment. Participants in the experimental arm had a personalized handout from the LCSDecTool summarizing their values and attitudes to prompt a shared decision-making discussion. When feasible, research coordinators went to the clinic with participants randomized to the experimental arm and offered providers the opportunity to access the LCSDecTool during the clinic visit. Following the clinic visit, participants completed post-visit (T1) surveys in-person at the clinic site or via telephone. Participants then completed 1-month (T2) and 3-month (T3) follow-up surveys by telephone. Study sites initially included the Philadelphia VA Medical Center and the West Haven VA Medical Center. In April of 2020, the West Haven VA Medical Center concluded active recruitment and the Clement J. Zablocki VA Medical Center in Milwaukee, WI, received Institutional Review Board (IRB) approval as an additional study site.

## Multi-level coordination and communication

### Immediate response to the pandemic: halting research

Due to rises in COVID-19 cases and the priority of controlling the pandemic, on 3/17/20, the ORD placed an administrative hold on most research activities, including in-person research. Pending research visits were canceled and recruitment for study visits halted. Information and direction regarding research procedures was newly emerging daily, so study coordinators were required to communicate effectively with both national leadership and local organizations to ensure they were following the most up-to-date regulations. Decisions regarding cancelations of scheduled appointments among previously recruited participants were made weekly through March, April, May, and June. On April 3, 2020, the team submitted a Note to File with the local Philadelphia VA IRB stating that the team was pausing enrollment of new patients and that they would continue with follow-up study visits by telephone as approved in the previous protocol.

### Coordination with clinical and diagnostic services: restarting research

Coordination across clinical and research services as well as across local and national levels of the Department of VA was necessary when considering restarting recruitment for the study. The National VA ORD allowed in-person research to resume on 6/8/2020, but gave local facilities flexibility to evaluate individual studies, set timelines, and institute study-specific and facility-wide restrictions. The Milwaukee VA resumed clinical visits using a phased approach, opening at 25% in mid-May, 50% on 7/13/20, and 75% on 9/14/20; research visits were allowed only if a potential participant was already coming for a clinical visit. As the Milwaukee VA resumed research visits before the Philadelphia VA, coordinators at both sites worked together to initiate recruitment in Milwaukee, including conducting chart reviews to identify potential participants and training the Milwaukee site coordinator to allow for an efficient start of recruitment at this new site.

The Philadelphia VA allowed in-person research to resume on 9/28/2020. Coordinators had to consider numerous factors in the resumption of research activities; in addition to the institution of safety procedures, coordination with clinical services was required. The purpose of the LCSDecTool is to help Veterans weigh the benefits and potential risks of LCS and support a shared decision-making discussion between patients and providers about LCS. However, this could only occur once the radiology department resumed LCS. As with much of routine care during the COVID-19 pandemic, cancer screening tests were temporarily discontinued as the facility prioritized urgent and acute care. The study team was in contact with facility LCS program leaders and did not resume study recruitment until the LCS program and radiology department were scheduling LCS tests.

Due to the study protocol’s inclusion of a baseline study visit directly preceding participants’ primary care appointments, the team had to communicate with leaders in the primary care clinics to coordinate recruitment activities with clinic procedures. Coordinators learned from providers and leadership that clinics were operating with reduced capacity with providers having limited in-person visits and primarily virtual ones. Furthermore, each clinic handled the restrictions differently. One community-based outpatient clinic (CBOC) was open to conducting in-person research while another within the same region was strictly opposed. These operational differences highlighted the variation in clinic sites and leadership response to the pandemic. To restart study recruitment and visits, it was necessary to communicate with leadership and follow guidelines across both study sites and their participating facilities.

## Flexibility and adaptability in research team roles and study procedures

### Adapting workforce activities and allocation of resources

While most research activities were halted, the study team adapted roles among their workforce and reconsidered the allocation of resources. Coordinators shifted efforts from recruitment to the collection of follow-up data, a process that was remote in the previously approved protocol. In anticipation of the resumption of research activities, coordinators at both active recruitment sites (Milwaukee and Philadelphia VAs) continued to identify and conduct chart review of patients potentially eligible for the study.

The study team also shifted their focus to data analysis and dissemination of study findings through writing scientific manuscripts from earlier phases of the study, including the development and usability evaluation of the LCSDecTool. With the disruption to the study, team members faced uncertainty about the future of the project and their own personal work lives, including their day-to-day tasks and job security, so the investigative team sought to find productive avenues for supporting the study without the direct work of participant recruitment.

During the pause in recruitment, the study team considered necessary changes to procedures in anticipation of restarting recruitment and in-person visits, including social distancing and use of personal protective equipment (PPE). Specifically, procedures required that research coordinators maintain 6 feet of distance from participants in a private room, provide and wear masks, and sanitize between visits. These added safety measures, in addition to the continued chart review, allowed coordinators to prepare for active recruitment when they received approval to resume research.

### Procedural changes

Once research was permitted to resume and it became apparent that most primary care clinics had shifted to telemedicine, the research team modified the study design to include virtual baseline visits. The research team had designed the LCSDecTool for use on multiple platforms, including a tablet, computer, and mobile phone. The Web-based tool could be opened through a link entered URL and a unique ID code. No personal health information was entered into the LCSDecTool. These design features allowed for remote use of the intervention. The research team submitted IRB modifications to allow for remote study visits, including approval for a verbal informed consent that could be administered by telephone. Prior to the pandemic, telephone oral consents were not commonly used in VA research studies. However, the restrictions on in-person research forced leadership to consider and accept alternative methods. The local IRBs had an expedited review process for adaptations to COVID-19 regulations and oral consent was approved in the lead site of Philadelphia on 11/4/2020 and in the Milwaukee site on 1/25/2021. When modifying the study protocol, the research team also anticipated that it may be more difficult to retain participants during the pandemic and, resultingly, increased compensation for completing follow-up surveys. Through this process, the research team gained experience in adapting study design and the use of virtual methods in healthcare settings.

### Adapting workflow to remote study visits

Several modifications in workflow were needed to adapt to remote study visits. Preparation time for baseline visits increased, as coordinators were required to mail study documents, including the oral informed consent form and surveys, to participants before telephone visits. All documents were mailed through the VA to ensure privacy and staff members followed proper pandemic safety protocols when handling them. While this method was effective for most participants, mailing did have some limitations. Disruptions to the US Postal Service and inclement weather during the winter caused some mailings to be delayed. Coordinators confirmed with participants that they received the study documents, and, if they did not, they continued to follow up with them. Coordinators also confirmed in advance that participants possessed technology and the Internet to access the LCSDecTool. Participants were only required to access the LCSDecTool during the initial baseline visit, so, if they lost Internet service after this visit, it did not affect their participation in the study. A notable change in study procedures was the use of an oral consent process. During the baseline tele-visit, coordinators reviewed the document to ensure that participants fully understood and agreed to participate in the study. The participant then had to access the LCSDecTool independently, with support provided by phone if needed. Coordinators observed the importance of modifying the study design to comply with both national and local VA guidelines implemented during the pandemic.

## Leveraging technology: opportunity and threats to study design

### Threat to internal and external study validity

Harnessing technology to adapt the study design threatened internal and external validity of study findings. Prior to the COVID-19 pandemic, all participants completed an in-person baseline, attended an in-person primary care appointment, and viewed the LCSDecTool or control intervention on an iPad. In the modified study design, participants had different experiences completing baseline surveys and using the LCSDecTool. Data analysis plans must be modified to evaluate the impact of these factors on outcomes. For example, difficulty with the technology could have led to frustration among participants and influenced their baseline survey responses. Furthermore, viewing the LCSDecTool on a phone compared to the larger screen of an iPad could impact the user experience. Also, coordinators were unable to directly observe and record participants’ progression through the LCSDecTool when viewed remotely, so field notes were limited to comments made or questions asked by participants during their use of the LCSDecTool. As a result of these limitations, navigation issues or other feedback may have been undetected or misinterpreted. When analyzing the data collected through field notes, it will be important to consider the threat this change in methods may pose to its reliability. In order to make in-person and virtual research visits more comparable in terms of observational data, future studies should include video technology, rather than only phone calls, when feasible.

The change in protocol allowing participants to use the LCSDecTool during both in-person and virtual research visits also provides an opportunity to study the effectiveness of the intervention in different settings. For example, the pre-pandemic protocol allowed primary care providers to view the LCSDecTool with patients during a clinic visit and observe its impact on provider-patient communication in the visit. During the pandemic, working with participants who viewed the LCSDecTool at home provided insight into how patients used the LCSDecTool independently. This gives the research team a unique opportunity to analyze and compare the LCSDecTool’s effectiveness in different settings. Given the barrier of time constraints in clinical visits, insights gained regarding patients’ ability to use the LCSDecTool at home could increase the scalability of this intervention.

### Challenges of technology and concern for equity

The use of technology may pose barriers to research participation and health equity. Some eligible patients could not participate because they lacked Internet access at home and, even when in-person research resumed, were not comfortable attending an in-person visit. This barrier may bias the study sample by excluding participants at lower income levels. The requirement for patients to have technology and Internet access to participate in research can exacerbate underrepresentation in research and health disparities among underserved groups.

In addition to technology and Internet access, computer literacy is needed to conduct a successful study visit remotely. Several participants with Internet access had difficulty accessing the study website. One participant was unfamiliar with using the Internet and had to seek his wife’s assistance in linking to the website. Another participant required several attempts to place the URL into the address bar and link to the website. It was challenging for coordinators to solve these technological issues remotely. Technical difficulties can cost valuable time for research visits that occur remotely, and patients that typically can have trouble using the Internet, such as older patients or those with lower formal education levels, may have difficulty participating in research.

It is important that clinical researchers consider how to combat inequities associated with virtual care provided through technology. When possible, researchers should aim to provide potential participants with the option of a safe in-person visit so those without the necessary technology are not excluded. If an intervention cannot be safely provided in-person, researchers should consider alternatives that do not rely on participants’ possession of technology at home. For example, researchers may mail printed materials or provide participants with the technological devices required for a particular study. The study team was not able to mail printed versions of this intervention because the LCSDecTool has several interactive features. Researchers must also design interventions to be user friendly among those with lower computer literacy and formal education levels. Finally, researchers should be trained to provide technical support to participants.

## Patient perspective

It is important to consider participants’ perspectives and preferences regarding study design. In a survey of Veteran patient and caregiver perspectives of clinical research during the pandemic, researchers found participants to be evenly divided regarding preference for a phone or in-person visit [10]. During recruitment outreach in our study, coordinators at both sites encountered potential participants expressing distress related to COVID-19. Several patients were apprehensive about spending more time at the clinic than was necessary; some cited this as a reason to decline participation. One patient stated that he was “not in a good mental place due to COVID-19” and was “not interested in participating in anything.” Another explained, “I would love to join your study, but I’m terrified of COVID-19 and just run in to my appointment and run back out.” The pandemic has also affected study participants’ willingness to receive cancer screening. One participant’s primary care provider conveyed in visit notes that “the patient refused any screening for lung cancer and postponed other desired screening due to COVID-19 fears.” The pandemic also impacts how patients respond to follow-up study visits. One participant was delayed in completing his 1-month follow-up because he had contracted COVID-19. These experiences with patients during the pandemic highlight the effect that stress and other emotional factors can have on individuals’ willingness to participate in research. Such factors exist outside of the pandemic, so it is important for researchers to develop strategies in which they may be able to recruit participants even during stressful events.

## Conclusions

The COVID-19 pandemic has required changes to study protocols in behavioral clinical trials. Some of these changes may persist for the duration of and even following the pandemic. Our experience emphasizes the importance of coordination and communication across study sites and across levels of an organization, the need for flexibility in research design and study team roles, and both the benefits and drawbacks of virtual methods, including impact on health equity and validity of study findings. Adapting clinical trials to the COVID-19 pandemic restrictions was challenging for both investigators and study staff, while the pandemic itself created barriers for patients’ ability and willingness to participate in research. However, the process led to growth and professional development for the study team. Coordinators gained collaborative experience as they worked as a team to resume the study between two sites and coordinate with clinical services. In addition, research coordinators refined their skills in modifying study design through the adjustments made to study protocols, adapting to both national and local VA guidelines implemented during the pandemic.

The COVID-19 pandemic may have lasting impacts on clinical research design and how patients participate in research studies moving forward. More nimble study designs that include virtual methods help remove barriers to participation, including risks of infection and transportation, time, and costs incurred by participants. While these benefits are significant, disparities in access to technology and computer literacy raise important issues of health equity in research and practice. Changes in the study design also raise issues with the internal and external validity of clinical trials impacted by the pandemic. Experience gained in adapting clinical research during the COVID-19 pandemic will inform efforts to anticipate and potentially overcome unforeseen obstacles to clinical research in the future.

## Data Availability

NA

## Abbreviations

CBOCs: Community-based outpatient clinics
HIPAA: Health Insurance Portability and Accountability Act
IRB: Institutional Review Board
LCS: Lung cancer screening
LCSDecTool: Lung Cancer Screening Decision-making tool
ORD: Office of Research and Development
PPE: Personal protective equipment
RCT: Randomized controlled trial
USPSTF: United States Preventive Services Task Force
VA: Veterans Affairs
